# Cancer patients participating in a lifestyle intervention during chemotherapy greatly over-report their physical activity level: a validation study

**DOI:** 10.1186/s13102-016-0035-z

**Published:** 2016-04-19

**Authors:** Karianne Vassbakk-Brovold, Christian Kersten, Liv Fegran, Odd Mjåland, Svein Mjåland, Stephen Seiler, Sveinung Berntsen

**Affiliations:** Oncologic Department, Southern Hospital Trust, Postbox 416, 4604 Kristiansand, Norway; Department of Health and Sport Science, University of Agder, Postbox 422, 4604 Kristiansand, Norway; Department of Health and Nursing Science, University of Agder, Postbox 422, 4604 Kristiansand, Norway; Surgical Department, Southern Hospital Trust, Postbox 416, 4604 Kristiansand, Norway

**Keywords:** Validation, Physical activity, Accelerometer, IPAQ, Cancer patient, Oncology

## Abstract

**Background:**

The short form of the International Physical Activity Questionnaire (IPAQ-sf) is a validated questionnaire used to assess physical activity (PA) in healthy adults and commonly used in both apparently healthy adults and cancer patients. However, the IPAQ-sf has not been previously validated in cancer patients undergoing oncologic treatment. The objective of the present study was to compare IPAQ-sf with objective measures of physical activity (PA) in cancer patients undergoing chemotherapy.

**Methods:**

The present study was part of a 12-month prospective individualized lifestyle intervention focusing on diet, PA, stress management and smoking cessation in 100 cancer patients undergoing chemotherapy. During the first two months of the lifestyle intervention, participants were wearing an activity monitor (SenseWear™ Armband (SWA)) for five consecutive days while receiving chemotherapy before completing the IPAQ-sf. From SWA, Moderate-to-Vigorous intensity PA (MVPA) in bouts ≥10 min was compared with self-reported MVPA from the IPAQ-sf. Analyses both included and excluded walking in MVPA from the IPAQ-sf. Results were extrapolated to a wearing time of seven days.

**Results:**

Sixty-six patients completed IPAQ-sf and wore the SWA over five days. Mean difference and limit of agreement between the IPAQ-sf and SWA including walking was 662 (±1719) min^.^wk^−1^. When analyzing time spent in the different intensity levels separately, IPAQ-sf reported significantly higher levels of moderate (602 min^.^wk^−1^, *p* = 0.001) and vigorous (60 min^.^wk^−1^, *p* = 0.001) PA compared to SWA.

**Conclusions:**

Cancer patients participating in a lifestyle intervention during chemotherapy reported 366 % higher MVPA level from the past seven days using IPAQ-sf compared to objective measures. The IPAQ-sf appears insufficient when assessing PA level in cancer patients undergoing oncologic treatment. Activity monitors or other objective tools should alternatively be considered, when assessing PA in this population.

**Electronic supplementary material:**

The online version of this article (doi:10.1186/s13102-016-0035-z) contains supplementary material, which is available to authorized users.

## Background

The number of new cancer cases is continuously rising and is estimated to grow from 12.7 million new cases in 2008 to more than 22 million new cases worldwide in 2030 [1, 2]. Parallel to this increase, the possibilities for surviving cancer has never been better with 32.6 million cancer survivors worldwide in 2012 [3]. Many cancer survivors are expected to return to normal and productive lives following their diagnosis. However, cancer and its treatments are often associated with long-term impairment of physical, mental and psychosocial health and survivors are at risk of developing co-morbidities [4, 5].

Physical activity (PA) is recommended as a strategy both during and after chemotherapy to manage treatment-related symptoms, prevent early and late co-morbidities, improve quality of life, increase the rate of chemotherapy completion and possibly extend overall and disease-specific survival in cancer patients [6–8]. Consequently, the American Cancer Society has provided a set of general PA recommendations for cancer patients and survivors. Accordingly, cancer patients should avoid inactivity, try to return to normal daily activities as soon as possible following diagnosis, and follow the general PA guidelines for aerobic and strength exercise. This recommendation is >150 min of moderate PA per week combined with strength training two days per week. Further, the importance of individualizing these PA recommendations to the patient’s condition and preferences is pointed out, and it is emphasized that cancer patients may need to exercise at a lower intensity and/or for a shorter duration during their treatment [6]. These global PA recommendations are largely based on self-reports of cancer patients’ PA levels [6].

The International Physical Activity Questionnaire (IPAQ) is a validated questionnaire developed to monitor self-reported PA levels in healthy adults [9], and the most commonly used self-report tool of PA worldwide [10, 11]. However, limitations of the IPAQ include its length, low compliance and difficulties in completing the questionnaire [12]. These difficulties may be of even greater magnitude for cancer patients experiencing disease and treatment-related side-effects like fatigue, loss of interest, and cognitive difficulties [13, 14] when undergoing chemotherapy. A short form of the IPAQ (IPAQ-sf) is therefore preferred and previously used in cancer patients [15], but has not been validated in cancer patients. Cancer is primarily a disease of the elderly [16], which can make the use of the IPAQ-sf challenging since the questionnaire is developed for adults aged 18–65 years [9]. Secondly, the IPAQ-sf defines moderate PA as activities that make you breathe somewhat harder than normal [17]. In this regard it is of major importance to be aware of the fact that cancer patients undergoing chemotherapy usually are fatigued, which may impair the patients’ perceived level and intensity of PA. A validation study of IPAQ-sf across 12 countries revealed large variations with respect to correlation between IPAQ and objective measures of PA by activity monitors [9]. Thus, it is likely that the accuracy of the IPAQ varies when different populations are assessed, dependent on the populations’ demographics, cultural backgrounds, physical fitness, level of physical functioning and disease status [12, 18]. The objective of the present study was therefore to compare time in Moderate-to-Vigorous intensity PA (MVPA) recorded with the short form of the IPAQ with activity directly quantified using SenseWear™ Armband (SWA) in cancer patients receiving chemotherapy with curative or palliative intent.

## Methods

The present validation study is part of the I CAN-study [19]; a 12-month prospective feasibility intervention with the aim to increase population based adherence to healthy lifestyle behaviors including diet, PA, mental stress management and smoking cessation. The intervention was delivered to 100 cancer patients undergoing chemotherapy with curative or palliative intent through: 1) a grouped start-up course with patients and nearest relatives, 2) an information binder with recommendations, recipes and tips on how to manage possible disease and treatment-related symptoms themselves, and 3) monthly counseling with a lifestyle supervisor with recommendations individualized to the patients’ abilities, barriers and preferences. The study is described in detail elsewhere [19].

### Participants

All cancer patients receiving chemotherapy for all cancer types, with either curative or palliative intent, at one oncology center in Kristiansand, Norway were considered for study participation against the following inclusion criteria: 1) age ≥ 18 years; 2) life expectancy ≥ 6 months; 3) Eastern Cooperative Oncology Group performance status (ECOG) ≤ 2; and 4) able to speak and read Norwegian. The only exclusion criterion was suspected anorexia cachexia syndrome. The study was conducted according to the guidelines of the Helsinki Declaration. The Regional Committee for Medical and Health Research Ethics, South-East approved the study (ref.no. 2012/1717/REK). Written informed consent was obtained from all patients before inclusion.

### Procedures

Medical and demographic characteristics were collected via self-report and medical records. These included date of birth, height (collected by the physicians before start-up of chemotherapy), tumor type (later categorized into 1 = breast cancer; 2 = colorectal cancer; 3 = prostate cancer; 4 = other cancer types), tumor stage (I-IV), ECOG (0–2), treatment intention (curative or palliative), marital status, cigarette smoking status and education level. Weight was measured to the nearest 0.5 kg (Mechanical scale, Seca 761, Birmingham, United Kingdom) and body mass index (BMI) was calculated by dividing weight (kg) by height (m) squared. Self-reported PA was assessed using the IPAQ-sf. Objective quantification of PA was acquired via the SWA, either in conjunction with the participants’ first or the second appointed visit in the I CAN-study. The participants had undergone chemotherapy from five to twelve weeks at this time point. Participants were instructed to wear the SWA for five consecutive days; both including work week and weekend days. Since the present study was part of a comprehensive lifestyle study, participants’ diet, mental stress, cigarette smoking and quality of life were assessed in addition to their PA level; a total of 59 questions.

#### Short form of the International Physical Activity Questionnaire (IPAQ-sf)

The IPAQ-sf questionnaire assesses PA in bouts of ≥ 10 min as part of leisure time, domestic and gardening activities, and work and transportation activities the past seven days. PA is classified as either Vigorous PA (VPA), Moderate PA (MPA) or as walking [17]. All walking was included to MPA, as proposed by Craig et al. [9]. Additionally, MPA minus time spent on walking is presented. Total PA was defined as the sum of time in MVPA.

#### SenseWear Armband

SenseWear™ Armband Pro_3_ and Mini (BodyMedia Inc. Pittsburgh, PA) has been shown valid compared to indirect calorimetry in cancer patients (underestimation of daily energy expenditure by 9%, *r* = 0.68; *p* < 0.01) [20] and doubly labeled water in healthy adults (underestimation of daily energy expenditure by 5%, *r* = 0.81; *p* < 0.01) [21]. The sensor array includes an accelerometer, heat flux-sensor, galvanic skin response sensor, skin temperature sensor and a near-body ambient temperature sensor [21]. The SWA was worn on the triceps muscle halfway between the acromion and olecranon processes of the upper arm, as recommended by the manufacturer. Participants were instructed to remove the SWA during water-based activities, such as swimming or bathing, as the monitor is not waterproof. Data were downloaded with software developed by the manufacturer (SenseWear Professional Research Software V.6.1 for Pro_3_ and V7.0 for Mini, algorithm V.2.2.4) after entering necessary demographic characteristics (sex, age, height, weight, smoking status).

The SWA was programmed to record PA in 1-min epochs. The cut-points defined MPA as 3–6 METs and VPA >6 METs in bouts ≥10 min. Total PA was defined as the sum of MVPA in bouts of ≥10 min. Min^.^wk^−1^ from the SWA for each participant was calculated by multiplication of mean MVPA min^.^day^−1^ by seven. Complete measurements required SWA wearing-time ≥19.2 h^.^day^−1^ on at least one day.

### Data analysis

Descriptive characteristics are presented as mean and standard deviation (SD). The PA data are presented as min^.^wk^−1^ in the present study. The mean difference (IPAQ-sf minus SWA) ± 1.96 SD was calculated according to Bland and Altman [22]. To test if the IPAQ-sf overestimated PA compared to SWA, we applied a Wilcoxon signed-rank test for the absolute values for each activity monitor. Differences are presented as means with 95 % Confidence Intervals (CI). Additionally, the percentage discrepancy between IPAQ-sf and the SWA was calculated for each individual within different intensity categories. A linear regression with MVPA from SWA as the independent variable and the difference between IPAQ-sf and SWA for MVPA as the dependent variable was applied to test for systematic over-reporting. Level of significance was set to 0.05. Statistical analysis was performed with SPSS statistical software version 22 (SPSS Inc., Chicago, IL, USA). A post hoc power analysis using G*Power [23] yielded a power of 0.99 to detect differences between means, based on an effect size of 0.5.

## Results

From the 100 I CAN participants, 16 participants dropped out before enrolment in the present validation study. The remaining 84 participants received a SWA and completed the IPAQ-sf. Of these, 18 were eliminated due to 1) SWA malfunction (*n* = 14) or 2) not sufficient wearing-time of the SWA (*n* = 4). In total 66 participants had valid registrations on the SWA and were included to the present validation study. There were no significant differences in self-reported MVPA between participants vs. non-participants at baseline (*p* = 0.414). Participants wore the SWA for an average of 3.6 days and 23.7 h^.^day^−1^ of all valid days. Demographic, medical and physical characteristics of the participants (*n* = 66) and non-participants (*n* = 34) are presented in Table 1.

**Table 1:** Characteristics of participants (*n* = 66) and non-participants (*n* = 34). Presented as frequencies and percentages in parenthesis unless otherwise stated*

	Participants		Non-participants		*P*-value
	*n* = 66	(%)	*n* = 34	(%)	
Age, mean (SD)*	59 (11)		62 (12)		.254
Height, mean (SD)*	169 (9)		172 (8)		.111
Weight, mean (SD)*	72 (14)		78 (16)		.085
BMI, mean (SD)*	25.2 (4.3)		26.1 (4.4)		.335
Waist circumference, mean (SD)*	91 (12)		96 (13)		.097
Sex					
- Men	18	(28)	12	(35)	.407
- Women	48	(72)	22	(65)	
Marital status					
- Married/living together	57	(86)	23	(68)	.027
- Single/divorced/widowed	9	(14)	11	(32)	
Education level^a^					
- High school or less	34	(52)	13	(41)	.279
- College/university	31	(48)	19	(59)	
Cigarette smoking					
- Smoker	8	(12)	5	(15)	.716
- Non-smoker	58	(88)	29	(85)	
ECOG					
- 0	49	(74)	29	(85)	.312
- 1	16	(24)	4	(12)	
- 2	1	(2)	1	(3)	
Treatment intention					
- Curative	44	(67)	17	(50)	.106
- Palliative	22	(33)	17	(50)	
Tumor stage					
- I	12	(18)	4	(12)	.564
- II	14	(21)	7	(20)	
- III	16	(24)	6	(18)	
- IV	24	(37)	17	(50)	
Diagnosis					
- Breast cancer	35	(53)	11	(32)	.036
- Colorectal cancer	22	(33)	10	(30)	
- Prostate cancer	2	(3)	2	(6)	
- Other	7	(11)	11	(32)	
Self-reported moderate-to-Vigorous PA, mean (SD)* (min^.^wk^−1^)	833 (989)		675 (694)		.414

^a^Missing value

*standard deviation

### Time in MPA, VPA and MVPA, including and excluding walking

**Fig. 1:**
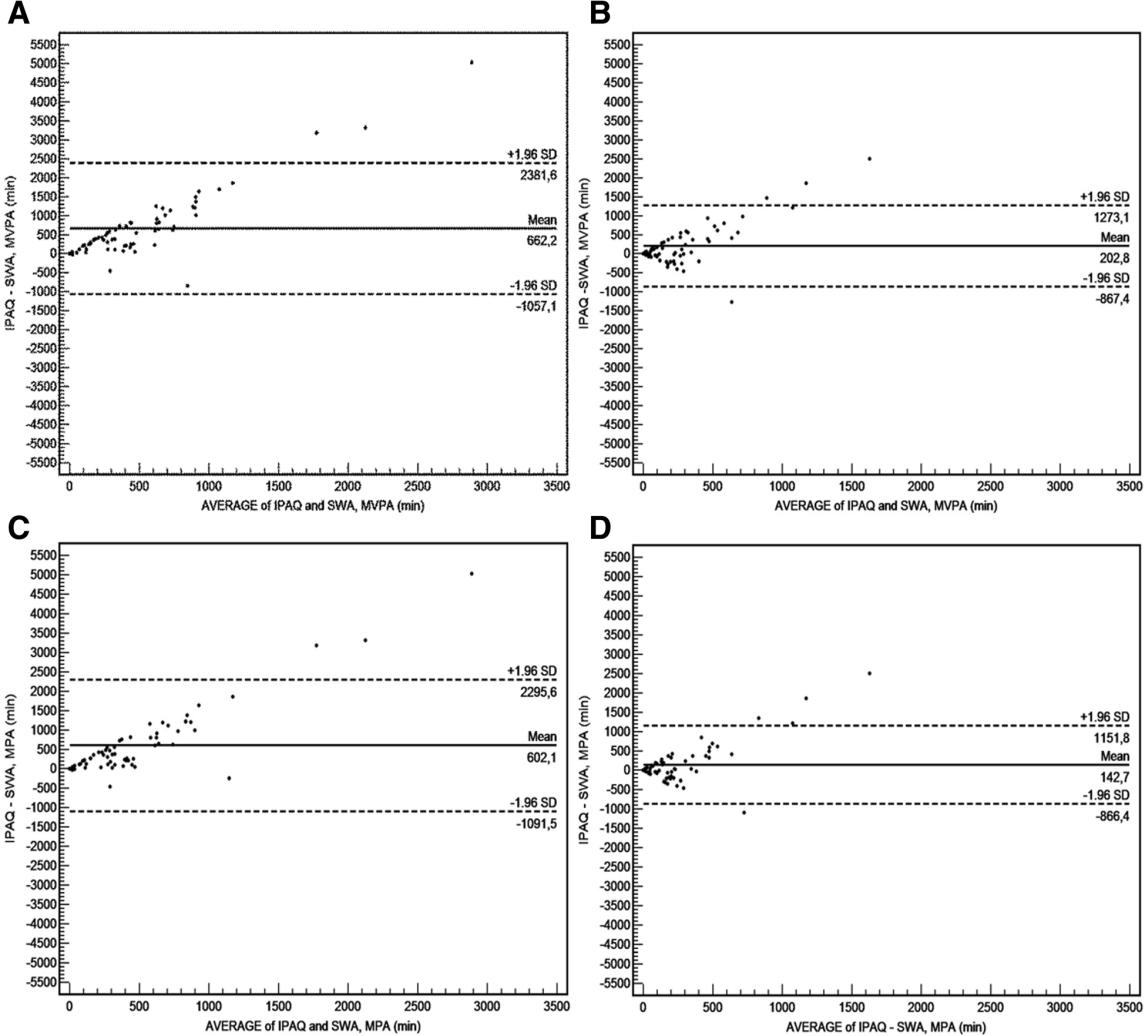
Bland-Altman plots depicting the mean differences (IPAQ-sf minus SenseWear Armband) for minutes spent in **a** Moderate-to-Vigorous intensity Physical Activity (MVPA) including walking, **b** MVPA excluding walking, **c** Moderate intensity PA (MPA) including walking and **d** MPA excluding walking. The solid line represents the mean, and the dashed lines represent the 1.96 SDs of the observations

The mean differences and limits of agreements between the IPAQ-sf and SWA from the Bland-Altman plots for time in MVPA were 662 (1719) min^.^wk^−1^ and 203 (1070) min^.^wk^−1^ with walking included and excluded in the analyses, respectively (Fig. 1a and b). Figure 1c and d depicts the mean differences and limits of agreements between the IPAQ-sf and the SWA from the Bland-Altman plots for time in MPA with walking included and excluded from the analyses; 602 (1694) min^.^wk^−1^ and 143 (1009) min^.^wk^−1^, respectively. Furthermore, Fig. 1 shows that several of the participants also under-reported their PA compared to the SWA (plots under the solid line). From the IPAQ-sf, 23 participants reported VPA during the last week. The SWA only identified three participants conducting VPA during the same seven-day period, and VPA is thus not depicted in a Bland-Altman plot. Linear regression revealed no significant systematic over-reporting; indicating those who had the highest levels of PA did not significantly over-report their PA levels the most. When comparing the min^.^wk^−1^ differences between IPAQ-sf and the MVPA recorded with SWA, analyses revealed a 366 % higher MVPA level reported on the IPAQ-sf compared to SWA (*p* = 0.001) (Table 2). After excluding walking from the analysis, the IPAQ-sf still reported statistically significant higher levels of MVPA; 112 % higher compared to the SWA (*p* = 0.007). When time in the different intensity categories were analyzed separately, IPAQ-sf reported significantly more time spent in MPA (602 min^.^wk^−1^, *p* = 0.001) and VPA (60 min^.^wk^−1^, *p* = 0.001) compared to SWA; 342 % and 1200 % more, respectively. Stratified analyses on mean differences between patients undergoing curative vs. palliative chemotherapy revealed no significant difference between the groups in over-report of MVPA (733 (443, 1023) vs. 521 (211, 832) min^.^wk^−1^, respectively, *p* = 0.395).

**Table 2:** Mean difference and 95 % Confidence Intervals (95 % CI) for PA obtained by the IPAQ-sf and SenseWear Armband data (*n* = 66)

	IPAQ-sf – SWA		*P*-value
	Mean difference (min^.^wk^−1^)	(95 % CI)	
Moderate PA			
- Walking included	602	(390, 815)	.001
- Walking excluded	143	(16, 269)	.107
- Walking vs. Moderate (IPAQ-sf vs. SWA)	283	(145, 421)	.001
Vigorous PA^a^	60	(28, 94)	.001
Moderate-to-Vigorous PA			
- Walking included	662	(447, 878)	.001
- Walking excluded	203	(69, 337)	.007

^a^Missing values

### Accommodation of PA guidelines

Participants were categorized into fulfilling the American Cancer Society PA guidelines of ≥150 min^.^wk^−1^ or not when comparing MVPA obtained from the IPAQ-sf vs. the SWA. Analyses revealed significant differences between the IPAQ-sf and the SWA when walking was included to the MVPA; IPAQ-sf identified 58 of the participants as meeting the PA guidelines vs. 32 by SWA (*p* = 0.001). After excluding walking from the analyses, 35 of the participants self-reported accommodating the PA guidelines (*p* = 0.532).

## Discussion

In the present study, cancer patients participating in a comprehensive lifestyle intervention while undergoing chemotherapy over-reported time in MVPA compared to SWA, when completing the IPAQ-sf. Specifically, these cancer patients over-reported their moderate-to-vigorous PA by nearly 100 min^.^day^−1^. No differences in over-reporting were observed between patients undergoing chemotherapy with curative or palliative intent.

Almost 90 % of the participants in the present study perceived themselves as meeting the PA guidelines of 150 min^.^wk^−1^ of MPA while less than 50 % actually met the PA guidelines according to the objective measures. Our findings are supported by previous studies, where more than half of healthy adults included perceived themselves as accommodating the PA guidelines [24, 25]. Objective measures in the same population revealed that only 15 % were reaching the guidelines [26]. The higher percentage of participants self-reporting accommodating the PA recommendations observed in the present study compared to previous studies [26] may be a result from drawing our participants from a lifestyle intervention focusing on the participants’ diet, mental stress and smoking cessation in addition to their PA. In addition to measuring the participants’ lifestyle behaviors, participants also received recommendations on how to maintain or adhere to a healthy lifestyle during their cancer treatment. It is a well-known phenomenon that individuals participating in a study often improve aspects of their behavior as a response to being studied or they believe they have improved more than what they actually have (the Hawthorne effect) [27–29]. In the present study, the IPAQ-sf identified significantly more participants as accommodating the PA guidelines of 150 min^.^wk^−1^ of MVPA vs. the SWA. Six of the participants reported from 420 to 840 min^.^wk^−1^ of MVPA, while less than 60 min^.^wk^−1^was registered on their SWA. These findings are not only statistical significant, but also of great clinical importance. Health care professionals should take these findings into consideration when delivering PA recommendations in this population and if using IPAQ-sf as an assessment tool in the clinic. In terms of this knowledge, individualized PA recommendations can be delivered with barriers such as fatigue, feeling sick, loss of interest and nausea, in mind [13, 30], so that cancer patients undergoing oncologic treatment can harvest the known health benefits from adhering to the developed PA guidelines [6].

Participants in the present validation study self-reported being in MVPA 662 min^.^wk^−1^ more than what was objectively registered by the SWA; an over-reporting that is supported in the literature [9, 31–33]. Of the 844 min^.^wk^−1^ MVPA reported on the IPAQ-sf in the present study, participants reported 459 min^.^wk^−1^ as walking activities. As reported previously, IPAQ-sf may over-report MPA because it includes walking at any intensity [33]. In the present study, IPAQ-sf over-reported time spent on walking by 283 min^.^wk^−1^ or 1.6 times compared to MPA obtained by the SWA. When including MPA from the IPAQ-sf in the analysis, IPAQ-sf over-reported walking by 602 min^.^wk^−1^ or 3.4 times compared to MPA data on the SWA. To include time spent on walking in the MVPA but not differentiate the intensity of the walking may be a potential source of over-reporting in cancer patients undergoing chemotherapy, and time spent on walking was thus both included and excluded to MVPA in the present study. A significant amount of the walking performed by these patients may be objectively registered as light intensity [34], while being experienced and self-reported as moderate [35]. The IPAQ-sf defines MPA as activities that make you breathe somewhat harder than normal [17]. Importantly, cancer patients dealing with disease- and treatment-related side-effects such as reduced physical capacity, fatigue, pain, depression and anxiety [36–39] may feel short of breath at a much lighter intensity than they have previously or compared to the population which the questionnaire is developed for. Consequently, the experienced side-effects are a great source of over-reporting since the patients might experience and report the PA as moderate, while the SWA assesses the PA as light intensity. This is important to have in mind when using self-reports in cancer patients; many PA self-reports, like the IPAQ-sf, are developed for use in a healthy population. Gil-Rey et al. [39] thus suggest cancer-specific PA guidelines to maximize the health benefits in this population.

The participants did not systematically over-report their PA levels when completing the IPAQ-sf; in other words, higher physical activity levels from the SWA was not associated with more over-reporting of MVPA reported from the IPAQ-sf. Our findings are in contrast to the findings of Johnson-Kozlow et al. [31], who revealed larger over-reporting in the breast cancer survivors who reported the highest PA levels on the long form of the IPAQ. Reasons for our conflicting findings may be the use of different accelerometer in the two studies and the use of the long versus the short form of the IPAQ in the study of Johnson-Kozlow and colleagues [31] and the present study, respectively. The long form of the IPAQ gives a total of 35 examples of PA across different activity domains, i.e. recreational sports, leisure time and housework PA. This provides many opportunities for “forward telescoping”; a recall bias occurring if the activities were recalled as taking place during the same seven-day period which is monitored, but actually took place previously [40]. Secondly, while the participants were undergoing chemotherapy in the present study and may not have been able to perform much vigorous PA, the participants in the study of Johnson-Kozlow et al. [31] were cancer survivors two years post diagnosis. Recall of vigorous PA is more likely to be subject to “forward telescoping” on PA self-reports, since these activities are often easier to remember due to strong, distinct physiological signals [40].

Importantly, IPAQ has been criticized for being complicated to complete [41] due to difficulties in remembering which activities they performed the past seven days, to distinguish the intensity of the different activities and last but not least trying to identify whether or not the activities lasted ≥ 10 min [42]. These aspects may be even harder to recognize for cancer patients experiencing disease- and treatment-related side effects such as pain, mental stress, cognitive difficulties and fatigue [13, 14, 37]. When comparing the IPAQ-sf to the SWA, activities lasting ≥ 10 min from the SWA was applied in the analyses, which may lead to great over-reporting if the activity lasted < 10 min. Thus, post hoc PA comparisons assessed by the IPAQ-sf and minute-by-minute SWA data were conducted in the present study (data not shown). Analyses revealed that IPAQ-sf still significantly over-reported VPA but IPAQ-sf now significantly under-reported MPA by 44 % compared to the SWA. The difficulties in completing the IPAQ-sf are reasonably clear. Importantly, there are currently no gold-standard for quantifying PA [43]; however, accelerometers are a precise and valid tool and thus commonly used in validating self-reports of PA [32]. Consequently, it is of concern that both the evidence regarding PA in cancer patients and the PA recommendations in this population is developed on the basis of self-reported PA data, which in turn has impact on the validity of those recommendations [6]. Another question that arises in this context is why self-reports only addresses PA in bouts >10 min, when there is rapidly growing evidence on health benefits in shorter bouts of PA [44, 45]. This is; however, an unexplored field in cancer patients, which needs further investigation, especially since activities of longer duration may be hard to complete for cancer patients with impaired physical capacity due to the oncologic treatment.

There are strengths and limitations of the present study. To our knowledge, this is the first time the IPAQ-sf has been compared to an objective PA monitor in cancer patients undergoing chemotherapy with either curative or palliative intent. One key aspect when designing the present study was to make the objective registrations feasible to the patients. Many different guidelines regarding days wearing the activity monitors are previously provided, with a minimum wearing time of four days recommended [46, 47]. One weakness of the present study is that only 66 % of the eligible patients were included, despite wearing the SWA for only five days, due to voluntary dropout, SWA malfunction or insufficient wearing time, leading to a higher inclusion of breast cancer patients who were either married or living together with someone compared to the total sample. These observations may indicate that a shorter wearing time is preferred in this population with regard to feasibility. Secondly, the participants were instructed to remove the SWA during water-based activities and activities such as swimming were thus not recorded. However, cancer patients at our clinic were advised to refrain from activities such as swimming in public pools due to reduced immune function and increased infection risk during chemotherapy. Further, the present study, as previous lifestyle interventions, is limited by including the healthier and fitter participants compared to the population from which they are drawn [19, 30]. Unfortunately, no Bland-Altman plot was calculated for VPA since only three of the participants had bouts of ≥10 min of VPA recorded on the SWA. Further, the present study is limited by the amount of questions asked. The present validation study was part of a comprehensive lifestyle intervention, which focused on the participants' diet, mental stress, smoking cessation and quality of life in addition to their PA level. The large amount of questions asked, perhaps in combination with side effects from the disease and its treatment, may have affected the accuracy of the participants’ answers.

## Conclusion

Based on our findings, cancer patients participating in a lifestyle intervention while undergoing chemotherapy grossly over-report their PA level from the past seven days when using the IPAQ-sf. Thus, the IPAQ-sf seems to be insufficient when assessing PA level in cancer patients undergoing oncologic treatment. Activity monitors or other objective tools should be considered in this population as an attempt to bridge the gap between how physical active the cancer patients perceive themselves and how physical active they actually are, in order to provide each patient with correct and individualized PA recommendations.

### Ethics approval and consent to participate

The Regional Committee for Medical and Health Research Ethics, South-East approved the study (ref.no. 2012/1717/REK). Written informed consent was obtained from all patients before inclusion.

### Consent for publication

Not applicable.

### Availability of data and materials

All relevant data is contained within the manuscript and the Additional file 1.

## Additional file

Additional file 1: Availability of data and materials. (XLSX 47 kb)

## Abbreviations

ECOG: Eastern Cooperative Oncology Group performance status
IPAQ-sf: international physical activity questionnaire, short form
METs: metabolic equivalent tasks
Min: minutes
Min^.^wk^−1^: minute per week
MPA: moderate physical activity
MVPA: moderate-to-vigorous physical activity
PA: physical activity
SWA: SenseWear Armband
VPA: vigorous physical activity
